# Postoperative cage migration and subsidence following TLIF surgery is not associated with bony fusion

**DOI:** 10.1038/s41598-023-38801-7

**Published:** 2023-08-03

**Authors:** Marcus Rickert, Peter Fennema, Diana Wehner, Tamim Rahim, Bernd Hölper, Michael Eichler, Marcus Makowski, Andrea Meurer, Marco Brenneis

**Affiliations:** 1 Spine Department, Schön Klinik Lorsch, Wilhelm Leuschner Strasse 10, Lorsch, Germany; 2 ARM Advanced Medical Research, Hofenstrasse 89b, 8708 Männedorf, Switzerland; 3 Wirbelsäulenzentrum Fulda/Main/Kinzig, Hailerer Straße 16, 63571 Gelnhausen, Germany; 4 Asklepios Klinik Wiesbaden GmbH, Geisenheimer Straße 10, 65197 Wiesbaden, Germany; 5 Department of Radiology, MRI TU Munich, Klinikum rechts der isar der TU München, Ismaninger Strasse 22, Munich, Germany; 6 Department of Orthopedics, Orthopadische Universitatsklinik Friedrichsheim gGmbH, Frankfurt am Main, Hessen Germany; 7 Department of Trauma and Orthopaedic Surgery, Goethe University Frankfurt, University Hospital, Theodor-Stern-Kai 7, 60590 Frankfurt am Main, Germany

**Keywords:** Spine structure, Neuroscience, Diseases

## Abstract

Pseudarthrosis following transforaminal interbody fusion (TLIF) is not infrequent. Although cage migration and subsidence are commonly regarded as evidence of the absence of solid fusion, there is still no evidence of the influence of cage migration and subsidence on fusion. This study aimed to evaluate cage migration and subsidence using computed tomography (CT) DICOM data following lumbar interbody fusion. The effects of cage migration and subsidence on fusion and clinical outcomes were also assessed. A postoperative CT data set of 67 patients treated with monosegmental TLIF was analyzed in terms of cage position. To assess the effects of cage migration and subsidence on fusion, 12-month postoperative CT scans were used to assess fusion status. Clinical evaluation included the visual analog scale for pain and the Oswestry Disability Index. Postoperative cage migration occurred in 85.1% of all patients, and cage subsidence was observed in 58.2%. Radiological signs of pseudarthrosis was observed in 7.5% of the patients Neither cage migration nor subsidence affected the clinical or radiographic outcomes. No correlation was found between clinical and radiographic outcomes. The incidence of cage migration was considerable. However, as cage migration and subsidence were not associated with bony fusion, their clinical significance was considered limited.

## Introduction

Spondylodesis using transforaminal interbody fusion (TLIF) is a common procedure for the treatment of lumbar degenerative diseases. Currently, the percentage of solid fusions is 90% in the literature^1–4^. Although the literature shows that clinical outcomes do not necessarily correlate with successful fusion^5,6^, bony fusion should be considered the true goal of instrumented spondylodesis.

Implant-associated changes, such as cage migration and subsidence, are also associated with the occurrence of pseudarthrosis^7–10^. However, the criteria for the assessment of cage migration and subsidence are very heterogeneously defined and are not based on uniform threshold values. In fact, in the context of cage migration, only the posterior positional change of the cage, referred to as retropulsion, with a cage overhang over the posterior edge of the vertebral body is often considered^8,10–15^, whereas the incidence and significance of minor positional changes of the cage are neglected. Although cage migration and subsidence are occasionally defined as proof of the absence of a solid fusion^16,17^, robust scientific evidence demonstrating the effects of cage migration and subsidence on osteogenesis is lacking.

This study aimed to assess the association between the occurrence of cage migration or subsidence and fusion. Whether cage migration or subsidence is associated with clinical outcomes following spondylodesis was also examined.

## Materials and methods

This retrospective pilot study assessed the 1-year radiological and clinical outcomes after TLIF. Ethics committee approval was obtained from the Ethics Committee of the State Chambers of Physicians of Hessen, Germany, prior to the study’s commencement, and all patients provided written informed consent. The study was performed in accordance with the Declaration of Helsinki and under the terms of relevant German legislation.

All adult patients who underwent monosegmental posteriorly instrumented spondylodesis surgery with implantation of the CarRLIF (LfC, Czerwieńsk, Poland) TLIF cage between January 2012 and December 2014 were invited to participate in the study. The exclusion criteria included patients who used an interbody cage system other than the CarRLIF, those with an active tumor or inflammatory disease of the spine (e.g., spondylitis, spondylodiscitis) and acute spinal trauma, and those with an image material not qualitatively usable for the evaluation (e.g., due to artifacts).

The complete radiological and clinical data of 67 patients were available at the baseline. These patients included 42 women (62.7%) and 25 men (37.3%). The mean age of the patients at the time of surgery was 58.4 ± 13.2 years (range, 24–86 years). The mean body mass index was 28.0 ± 5.0 kg/m^2^ (range, 19.5–43.2 kg/m^2^). Thirty-eight patients (56.7%) had no previous surgeries in the corresponding surgical segment or in any of the adjacent segments. The remaining 29 patients (43.3%) had undergone at least one prior decompression in the operated segment. Eleven patients (16.4%) had multiple decompressions of the surgical segment, and 11 patients (16.4%) had at least one decompression in a subsequent segment. The indications for posterior spondylodesis using the TLIF technique were instabilities of the lumbar spine (degenerative spondylolisthesis, spondylolisthesis vera), lumbar deformities with spinal stenosis, postnucleotomy syndrome, and osteochondrosis of the lumbar spine as diagnosed on conventional standing anteroposterior and lateral radiography, flexion–extension radiographs^18^, and MRI. The operated segments included L2–L3 in two patients, L3–L4 in seven patients, L4–L5 in 39 patients, and L5–S1 in 19 patients. All patients underwent 3–6 months of unsuccessful conservative treatment prior to surgery.

The mini-open TLIF technique is performed through a bilateral paramedian incision according to Wiltse^19^. After opening the fascia and the transmuscular blunt dissection to the pedicle entry point, the pedicle screws are inserted using the mini-open technique under fluoroscopic control and connected using bilaterally inserted rods. A predistraction of the segment is applied. By subperiosteally pushing off the autochthonous back muscles from the spinous process and the hemilamina, the interlaminar window is then visualized. The segment is decompressed through a hemilaminectomy. The intervertebral foramen is opened through a unilateral resection of the facet joint, and the disc space is exposed. After the complete excision of the disc and the careful preparation of the inferior and superior endplates, the disc space is further distracted to restore the original disc height. To select the cage size, image intensifier-controlled trial cages are first inserted. The cage is then inserted under fluoroscopic control and finally positioned in the anterior third of the intervertebral space. Due to the particular design of the cage used in the present study, the additional insertion of bone or a bone substitute material is not necessary. This is followed by the compression of the segment, securing the screw–rod system using closure caps. Finally, the wound is closed stepwise following the anatomical layers. Postoperatively, patients did not receive an orthosis or corset. First physiotherapy was prescribed 6 weeks following the operation.

Computed tomography (CT) of the lumbar spine was performed within the first postoperative week after spondylodesis surgery to assess the position and implant location. To rule out early implant failure during the patient’s healing process, native radiological imaging of the lumbar spine was performed after 6 weeks. Native radiological imaging consisted of an assessment of implant location, loss of disc height, and loss of angular correction. At the final examination after 1 year, a new CT of the lumbar spine was performed to examine any evidence of segmental fusion, implant loosening/fracture, stress fracture, or instability in the index segment.

**Figure 1:**
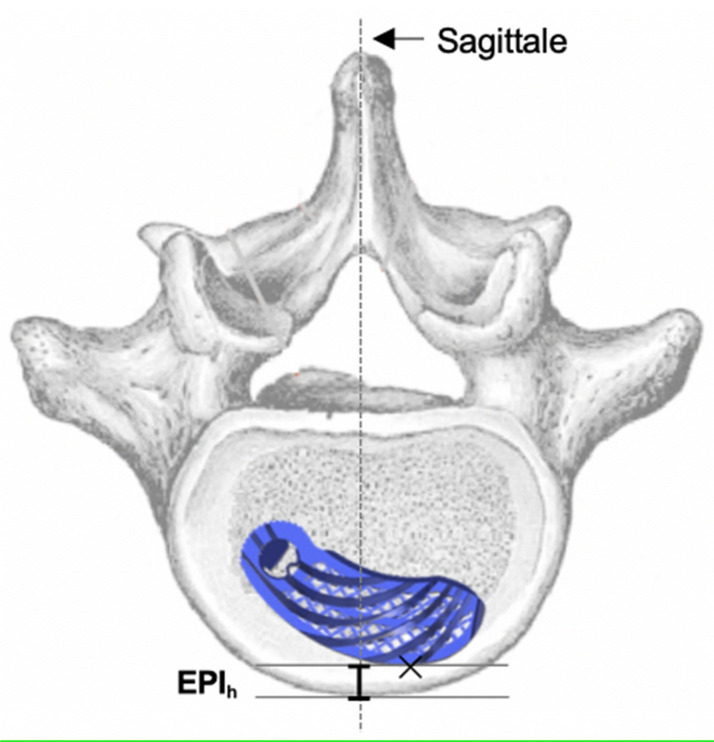
EPIh: Distance between the implant and the frontal part of the vertebral body.

**Figure 2:**
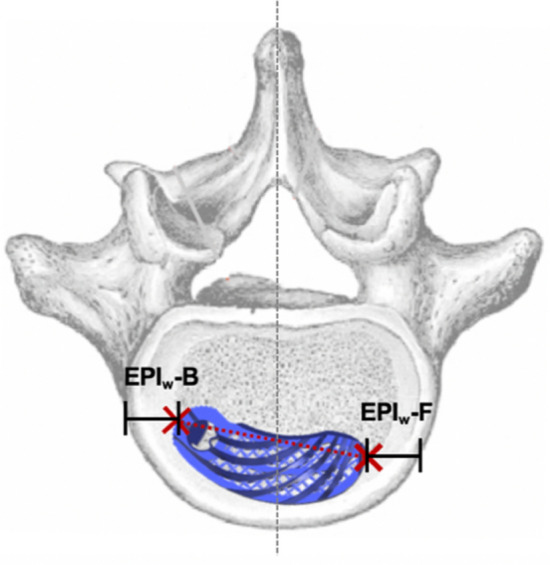
EPIw-B and EPIw-F: Width (w) between Endplate (EP) and Implant (I), laterally ipsilateral and contralateral.

**Figure 3:**
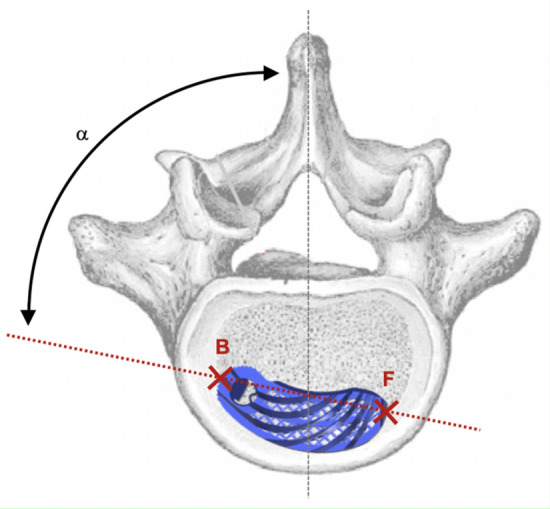
Implant axis in the axial plane.

**Figure 4:**
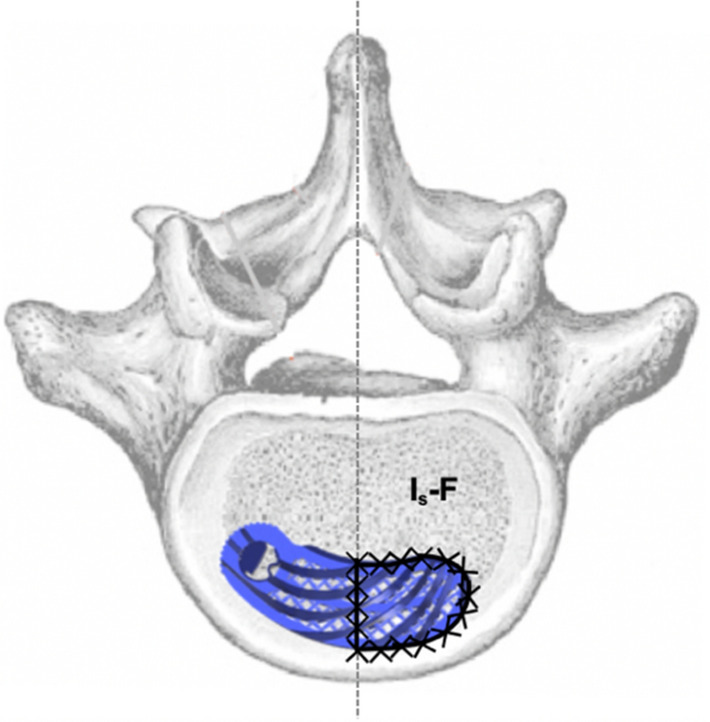
Surface measurement of the endplate on the left (I_s_-B) and right (I_s_-F) side of the median.

**Figure 5:**
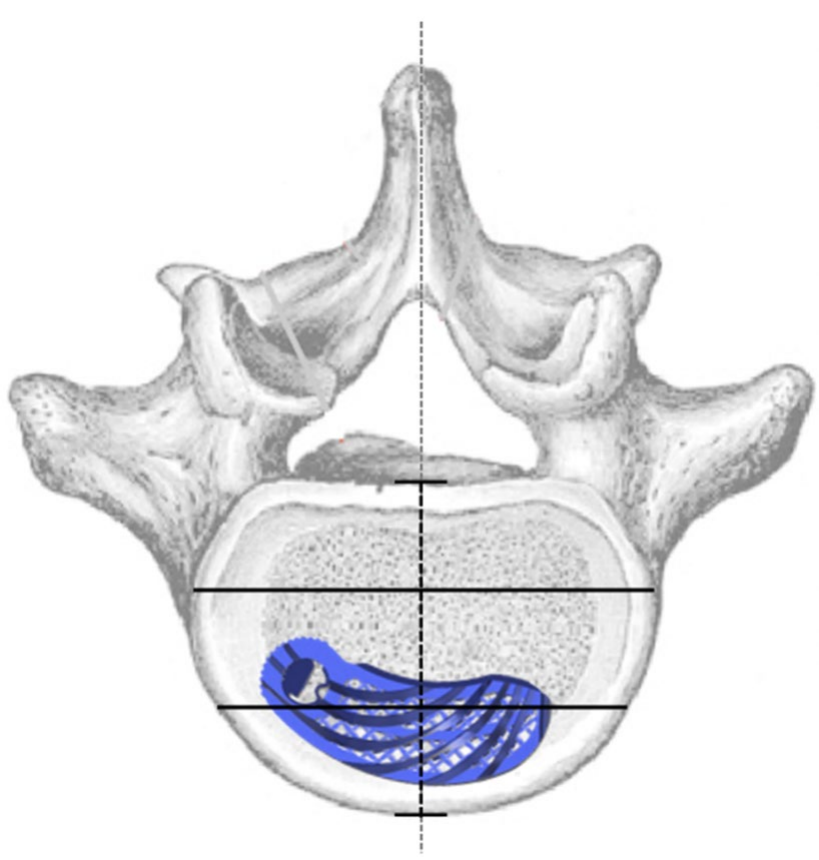
Qualitative appraisal of cage position. For this purpose, the endplate was divided into three areas (ventral, central, and dorsal) based on its depth, and the area with the largest contact surface of the cage was determined.

VGStudio Max software (version 2.1, Volume Graphics GmbH, Heidelberg, Germany) was used to analyze the cage position. Based on the vertebral body and cage-specific parameters, six measurement variables were defined to analyze the implant position in the axial sectional plane (Figs. 1, 2, 3, 4, 5). According to the developer of the software, the measurement accuracy varies between 0.3 and 1.0 mm, depending on the imaging quality.

For each measurement, the two CT data sets of a patient were imported into the standard DICOM-based software. Then, an axial reconstruction aligned parallel to the intervertebral space of the surgical segment was calculated from the imported data to determine identical measurement positions in the surgical segment.

For cage migration, the measured values determined in the postoperative CT scans were pairwise compared for each patient. Migration was classified as none, minimal, or substantial in accordance with the criteria in Table 1. Subsidence into adjacent vertebral bodies was assessed in accordance with the criteria in Table 2. The presence of fusion status was based on the Bridwell^20^ and Eck^21^ criteria in the CT scan (Table 3). The anterior and posterior columns were each considered in isolation. If grade I or II fusion was present, fusion could be assumed to be certain or probable. If grade III or IV was present, fusion was unlikely or excluded. The CT scan evaluation was performed by two senior spine surgeons, and fusion was determined based on consensus.

**Table 1:** Criteria for the evaluation of migration.

Category	Criterion
No migration	Δ EPI_h_ < 1 mm, AND Δ EPI_w_-F < 1 mm, AND Δ EPI_w_-B < 1 mm, AND Δ α < 3°
Minimal migration	Δ EPI_h_ ≥ 1 < 3 mm, OR Δ EPI_w_-F ≥ 1 < 3 mm, OR Δ EPI_w_-B ≥ 1 < 3 mm, OR Δ α ≥ 3 < 10°
Significant migration	Δ EPI_h_ ≥ 3 mm, OR Δ EPI_w_-F ≥ 3 mm, OR Δ EPI_w_-B ≥ 3 mm, OR Δ α ≥ 10°

**Table 2:** Measured variables for the analysis of subsidence.

Variable	Description
SA-U [mm]	Magnitude of the implant’s subsidence (S) into the anterior (A) part of the upper (U) vertebra
SP-U [mm]	Magnitude of the implant’s subsidence (S) into the posterior (P) part of the upper (U) vertebra
SA-L [mm]	Magnitude of the implant’s subsidence (S) into the anterior (A) part of the lower (L) vertebra
SP-L [mm]	Magnitude of the implant’s subsidence (S) into the posterior (P) part of the lower (L) vertebra

**Table 3:** Fusion criteria according to Bridwell^20^ and Eck^21^.

	Fusion degree	Fusion criteria
Ventral column	Grade I	Fusion with remodeling and trabeculae present
	Grade II	Graft intact, not fully remodeled and incorporated, but no lucency present
	Grade III	Graft intact, lucency present between implant and endplate
	Grade IV	Fusion absent
Dorsal column	Grade I	Bilaterally fused facet joints and transverse processes
	Grade II	Unilateral fusion, assessment on the opposite side more difficult
	Grade III	Possible lucency or bony defect in the fusion region
	Grade IV	Fusion absent, signs of material fatigue

Preoperatively, postoperatively, after 6 weeks, and after 12 months, the patients were interviewed by the surgeon about their condition and underwent a comprehensive neurological examination including a lower limbs assessment of the sensory and motor system. Each patient was invited to complete the back and leg pain visual analog scale (VAS, with a scale graduation of 0–10), and the Oswestry Disability Index (ODI) questionnaire^22^. The ODI is a tool to quantify a patient’s functional disability. A score from 0 to 4 indicates no disability, from 5 to 14 mild disability, from 15 to 24 moderate disability, from 25 to 34 severe disability, and from 35 to 50 completely disabled^23^.

Statistical analyses were performed using BiAS (University Hospital Frankfurt, Germany). Continuous variables were evaluated using the means, and ordinally scaled variables were evaluated using medians. The Wilcoxon signed-rank test, Friedman’s test, Fisher’s exact test, and Jonckheere–Terpstra’s test were used as additional statistical test procedures in the analysis of the radiological and clinical variables. The significance level alpha was set to 0.05 for all statistical tests.

## Results

Thirty-two cages (47.8%) were assigned to the ventral area and 35 cages (52.2%) to the central area of the endplate 1 year postoperatively. None of the cages were located in the dorsal portion of the endplate. Ten patients (14.9%) showed no cage migration after 1 year (Table 4). Minimal cage migration was detectable in 41 patients (61.2%). Another 16 patients (23.9%) showed significant cage migration at the end of the first postoperative year. Overall, cage migration was detectable in 85.1% of the patients. Retropulsion of the cage into the spinal canal with crossing of the posterior edge of the vertebral body did not occur.

**Table 4:** Cage migration.

Variable	Mean ± SD (range)	< 1 mm (n) (n [%])	≥ 1 < 3 mm (n [%])	≥ 3 mm (n) (n [%])
ΔEPI_h_ [mm]	− 0.98 ± 1.39 (− 5.39 to 2.02)	33 (49.3%)	28 (41.8%)	6 (9%)
ΔEPI_w_-F [mm]	− 0.82 ± 1.58	30 (44.8%)	28 (41.8%)	9 (13.4%)
ΔEPI_w_-B [mm]	0.05 ± 1.62 (− 4.28 to 4.60)	34 (50.7%)	30 (44.8%)	3 (4.5%)
Δa [°]	2.12 ± 5.04 (− 14.1 to 17.7)	43 (64.2%)	17 (25.4%)	7 (10.4%)

*ΔEPI*_*h*_ distance h between the Endplate (EP) and Implant (I), *EPIw-B* distance w between Endplate (EP) and Implant (I) in terms of the back (B) part of the Implant, *EPIw-F* width (w) between Endplate (EP) and Implant (I) in terms of the front (F) part of the Implant, *Δa* axial alignment of the implant, *SD* standard deviation.

In 28 patients (58.2%), no subsidence of the cage into the adjacent inferior or superior endplate occurred within the first postoperative year (Table 5). Another 22 (32.8%) patients showed minimal cage subsidence, and 17 (25.4%) patients showed significant cage subsidence.

**Table 5:** Cage subsidence.

Variable	Mean ± SD (range)	< 1 mm (n [%])	≥ 1 < 3 mm (n [%])	≥ 3 mm (n) (n [%])
ΔSA-U [mm]	− 1.02 ± 1.36 (− 4.22 to 0.00)	42 (62.7%)	18 (26.9%)	7 (10.4%)
ΔSP-U [mm]	− 1.10 ± 1.36 (− 4.92 to 0.00)	37 (55.2%)	22 (32.8%)	8 (11.9%)
Δ SA-L [mm]	− 0.84 ± 1.32 (− 5.56 to 0.00)	44 (65.7%)	16 (23.9%)	7 (10.4%)
Δ SP-L [mm]	− 0.57 ± 1.03 (4.29 to 0.00)	51 (76.1%)	14 (20.9%)	2 (3.0%)

*ΔSA-U* subsidence of the cage into the anterior part of the superior endplate, *ΔSA-U* anterior part of the inferior endplate, *ΔSA-L* anterior part of the inferior endplate, *ΔSP-L* posterior part of the inferior endplate, *SD* standard deviation.

The inferior endplate was more frequently affected by subsidence than the superior endplate (49.3% vs. 37.3%). In addition, the mean subsidence into the inferior endplate was 1.06 mm, which was greater than the mean subsidence into the superior endplate (0.71 mm). Subsidence of the posterior implant portion into the adjacent baseplate occurred most frequently (n = 30, 44.8%). The maximum subsidence was 5.56 mm.

Radiological fusion assessment of the anterior column demonstrated certain (grade I) or probable (grade II) fusion in 60 patients (89.6%). In another seven patients (10.4%), anterior fusion was considered unlikely (grade III) or excluded (grade IV).

With respect to the posterior column of motion segments, the degree of fusion was considered certain (grade I) or probable (grade II) in 62 patients (92.5%). In five patients (7.5%), fusion of the facet joints and transverse processes was unlikely (grade III) or excluded (grade IV).

Only one of the patients studied developed screw loosening in the first postoperative year. This patient also had significant cage migration, subsidence, and symptomatic pseudarthrosis with consecutive instability, which required revision surgery. Thus, the revision rate was 1.5%. The clinical results are summarized in Table 6.

**Table 6:** Clinical outcome.

	Preoperative	6 weeks	1 year
ODI	59.6 ± 9.6 (40–82)	32.8 ± 12.7 (10–71)	21.2 ± 16.8 (0–73)
VAS back pain	7.3 ± 1.7 (4–10)	3.0 ± 1.6 (0–8)	2.2 ± 2.0 (0–8)
VAS leg	7.4 ± 1.8 (0–10)	2.2 ± 1.9 (0–8)	1.3 ± 2.1 (0–9)

Presented as mean ± standard deviation (range). *ODI* Oswestry Disability Index, *VAS* visual analog scale.

No significant association was found between the fusion grades (grade I–IV) and the clinical outcome of the patients after 1 year (p-value (ODI) = 0.33, p-value (VAS back pain) = 0.38, p-value (VAS leg pain) = 0.32).

No statistically significant associations were found between cage migration and fusion (p = 0.21) (Table 7) or between cage subsidence and fusion (p = 0.66) (Table 8). A statistically significant association was found between cage migration and patient age (dichotomized around 60 years) (p = 0.026). Cage migration occurred in 44.1% of those under 60 years of age (i.e., 15 out of 34) and in 72.7% of those over 60 years (i.e., 24 out of 33). Among the cases, 20 out of 32 patients (62.5%) with a cage position in the anterior area of the endplate showed cage subsidence. Conversely, 27 out of 35 cases (77.1%) with a central cage position showed cage subsidence. This association did not reach the level of significance (p = 0.191).

**Table 7:** Fusion status by cage migration.

Migration	Fusion degree			
	Grade I	Grade II	Grade III	Grade IV
No migration	6 (9.0%)	4 (6.0%)	0 (0.0%)	0 (0.0%)
Minimal migration	33 (49.3%)	4 (6.0%)	0 (0.0%)	4 (6.0%)
Significant migration	12 (17.9%)	3 (4.5%)	0 (0.0%)	1 (7.5%)

Presented as number of observations (percentage of study population).

**Table 8:** Fusion status by cage subsidence.

Subsidence	Fusion degree			
	Grade I	Grade II	Grade III	Grade IV
No subsidence	21 (31.3%)	5 (7.5%)	0 (0.0%)	2 (3.0%)
Minimal subsidence	16 (23.9%)	5 (7.5%)	0 (0.0%)	1 (1.5%)
Significant subsidence	14 (20.9%)	1 (1.5%)	0 (0.0%)	2 (3.0%)

Presented as number of observations (percentage of study population).

## Discussion

The incidence of cage migration varies from 0.8 to 23% in the literature^10,11^, with the majority of studies investigating cage retropulsion with an overhang of the posterior edge of the vertebral body. In the present study, minimal and significant cage migration were detectable in 61.2% and 23.9% of the patients, respectively, resulting in an overall migration rate of 85.1%. Retropulsion of the cage was not detectable in any case.

The significantly higher migration rate in this study compared to the literature is considered to be due to the accuracy of the measurement method. We assert that minimal cage migration can be understood as a common phenomenon after interbody fusions, and it has received little attention in the scientific analysis of migration behavior. The results of the current study further demonstrate that cage migration is not equivalent to cage retropulsion.

Complications such as neural compression and pseudarthrosis are common in association with cage retropulsion. This can also be seen in the revision rate, which has been reported in the literature as 33.3–75%^10,14^.

In the present study, the presence of cage migration was not associated with the clinical scores (ODI, VAS, analgesic requirement) or radiographic fusion evaluation. The revision rate of the patient group with cage migration was low at 1.5%. It is possible that minor and clinically asymptomatic cage migration is not an expression of persistent segmental instability but rather an indication of an increasing incorporation of the cage in the postoperative course and thus a component of the bony fusion process.

Most studies have described cage migration in the posterior direction^24,25^. The results of the present study suggest that cage migration often occurs simultaneously in multiple directions. In the anterior–posterior direction, an almost exclusively posterior cage position change was observed.

With regard to posterior cage migration and retropulsion, numerous potential risk factors have already been identified. One study reported an association with a preoperative high disc space^10^. Other studies have found that undersized cages are a risk factor for posterior cage migration^15,26^. From a biomechanical perspective, this is caused by the insufficient restoration of tensile stress to the annulus fibrosus and the ligamentous apparatus—a factor that contributes significantly to the primary stability of cages^27,28^.

The positioning of the cage within the disc space continues to be important for primary stability. Various biomechanical studies have shown that segmental stability increases anteriorly in a position-dependent manner, and this can be considered a consequence of a greater distance between the cage and the center of rotation^29–32^. Conversely, others have demonstrated an increased risk of migration in association with a posterior cage position^7,26^.

In the present study, the effects of cage position on migration were also examined. No significant correlation was observed. However, in contrast to the results in the literature, all cages were positioned anteriorly or centrally and not in the posterior region of the endplate.

The pattern of cage migration or retropulsion can be narrowed down to an average of 1–4 months postoperatively^10,11,14,15,33^. Consequently, the 1-year follow-up interval, as in the current study, can be considered appropriate but does not allow a conclusion to be drawn regarding the cage migration pattern over time.

To further develop standardized follow-up concepts for postoperative mobilization after lumbar interbody fusion, studies are needed to examine the progression of cage migration and subsidence over time.

The reconstruction of the original intervertebral space height to restore physiologic lumbar lordosis and the width of the neuroforamina is among the major goals of interbody lumbar fusions. Bony fusion should also preserve the position of the segment in the long term^34–36^. The most common cause of secondary loss of correction is the subsidence of the cage into the adjacent endplate. Within the first eight postoperative months, there was again a loss of intervertebral space height to an average of 13.2 mm. From > 2 mm, the loss of correction was considered subsidence. Consequently, the subsidence rate was 76.7%^37^.

In the present study, the incidence of cage subsidence at 1 year was 58.2%. As was the case with cage migration, subsidence was not shown to have a negative effect on clinical outcomes or the success of bony fusion, consistent with the literature^37–39^.

In the evaluation of the radiological results of this study, the effect of cage position on subsidence was confirmed. The subsidence rate of the group with a central cage position was more than twice as high (77.14% vs. 37.5%) as that of the group with an anterior cage position. Anatomically, this position-dependent subsidence behavior is explained by the inhomogeneous nature of the endplate, the thickness of which increases from the center toward the periphery^40–43^. Therefore, the endplate exhibited the highest compressive strength in the region of its cortical rim.

The patients in the present study showed an age-related increase in cage subsidence. This observation may reflect age-related changes in bone quality and an increasing prevalence of osteoporosis, as previously reported in the literature^38,44^. However, because data on the bone density of the patients are not available in the current study, a relationship with osteoporosis can only be hypothesized.

The size and shape of the cage are also factors to be considered^45–47^. To reduce the risk of subsidence through optimal load distribution, a cage with the largest possible bearing surface is recommended. Finally, another surgical factor to consider with respect to potential subsidence is the extent of endplate preparation as part of the disc excision procedure^48^.

In this context, the increased incidence of subsidence of the posterior implant portion into the endplate in the present study suggests uneven support and higher pressure loading in the peripheral region of the cage. As the posterior implant portion was also located further centrally, subsidence in this area was also favored. Indications of increased subsidence as a result of endplate injury could be ruled out by measurement in the first postoperative CT.

Furthermore, the base plate was generally more frequently affected by subsidence than the upper endplate (49.3% vs. 37.3%). However, this result has not been confirmed in the literature. Studies that have differentiated between the base and the upper endplate in the analysis of subsidence behavior usually showed subsidence of the cage into the upper endplate^37,49–51^. A possible explanation for the different results in the present study is the observation that subsidence into the inferior endplate occurred more frequently in association with an incomplete reduction of the anteriorly slipped vertebral body. The contact surface of the cage on the inferior endplate was more centrally located when the slipped vertebra was not fully corrected compared to the endplate. This observation again confirms the position-dependent subsidence risk of the cage and emphasizes the importance of reducing the slipped vertebra. In cases in which a complete correction of translational malalignment is not possible intraoperatively, the observation in this study may provide strategic guidance. Here, a more anterior positioning of the cage is recommended to ensure good support by the anterior apophyseal ring of the upper vertebra or a far lateral positioning of the cage in the lateral part of the apophyseal ring to reduce the risk of the cage subsiding into the upper endplate.

The evaluation of fusion status has been the subject of numerous studies. Nevertheless, the comparability of individual studies is generally problematic, as there is no consensus on the scientific consideration of fusion status with regard to the imaging techniques and fusion criteria to be applied^52,53^. CT is the most sensitive method in the evaluation of solid fusion and the detection of pseudarthrosis^54^. The main criteria for a successful interbody or posterolateral fusion are evidence of trabecular bone bridges between the endplates or articular and transverse processes and the absence of lysis fringes in the fusion region. Conversely, signs of material fatigue (screw loosening or fracture) are considered indirect evidence of non-fusion. The absence of migration and subsidence of the implant can be considered a fusion criterion^16^.

The approach of standardizing fusion evaluation by measuring cage migration or subsidence was further investigated in the present study. No significant correlation between cage migration or subsidence and fusion outcomes was found. Only one of the five patients with non-fusion showed significant cage migration, and two patients showed significant cage subsidence at the same time. Therefore, the analysis of migration and subsidence behavior is not important for the evaluation of interbody fusion.

The patients in this study had a uniform radiologic follow-up through CT 12 months postoperatively. The fusion grading established by Bridwell^20^ and Eck^21^ was used to evaluate fusion status. The fusion rate at 12 months was 92.5%; that is, 62 of the 67 patients showed grade I (n = 51) or grade II (n = 11) fusion of the anterior and/or posterior columns.

Comparably high fusion rates are found in the literature, with fusion rates of 80–100% depending on the implants used, imaging techniques, and fusion criteria^4,54–59^.

In conclusion, the incidence of cage migration was considerable. However, as cage migration and subsidence were not associated with bony fusion, their clinical significance was considered limited.

## Data Availability

The data that support the findings of this study are available on request from the corresponding author, M.R.
